# Psychic Income Associated With Shanghai Tennis Masters and Residents’ Attitude

**DOI:** 10.3389/fpsyg.2021.666777

**Published:** 2022-01-17

**Authors:** Fengyun Zhang, Dongfeng Liu, Daniel Plumley, Mengyan Chai

**Affiliations:** ^1^School of Economics and Management, Shanghai University of Sport, Shanghai, China; ^2^Sheffield Business School, Sheffield Hallam University, Sheffield, United Kingdom

**Keywords:** residents, psychic income, involvement, attitude, sports events

## Abstract

Using Shanghai Tennis Masters as an example, this study seeks to explore the psychic income associated with major sports events hosting and whether the psychic income would predict the attitudes of local residents toward events hosting. In addition, the moderating effect of sport involvement on the relationship between psychic income and attitude is also tested. In this study, a questionnaire survey is adopted. The structured questionnaire was developed based on 4 parts, including the demographics of the residents, involvement in the sport event, psychic income from the sport event, and their attitudes toward the sports event, there were 47 items in total. Data were collected from the local residents of Shanghai (including 16 districts or counties), as a result, 1,302 valid questionnaires were collected. A series of statistical analyses were conducted by using SPSS25.0 and AMOS 24.0 to examine the reliability and validity of the scales and to test the hypotheses. The results showed that the event has brought a significant level of psychic income to the local community, and the perceived psychic income would predict the attitudes of the residents toward the event hosting. The moderating effect of sports involvement on the relationship between psychic income and attitude is also confirmed.

## Introduction

Sports events are considered to have a significant impact on sustainable community development in the host country, especially economic, social, and cultural development (Thomson et al., 2019). The success of a mega sport event relies heavily on the local community recognizing the benefits of hosting the sport event (Kim and Kaplanidou, 2019). As one of the major stakeholders, the attitude of host city residents is considered key for a successful hosting of a major sports event and hence its sustainability (Kim et al., 2015; Lian and Huang, 2017).

It can be concluded from the existing literature that the attitudes of the residents toward the sports event are affected by many variables such as their involvement, affect, sense of belonging to the community, and demographics, etc. (Bee and Havitz, 2010; Liu et al., 2014; Zhang, 2015; Mao and Huang, 2016; Han et al., 2018; Zhang and Huang, 2018; Thomson et al., 2020). Among all these variables, psychic income reflects the residents’ psychological perception of hosting a sport event, especially their positive psychology and affect of the sport event. As an important part of psychological perception, affect can have a significant impact on the individual’s attitude formation and behavior change (Bee and Havitz, 2010; Liu et al., 2014; Zhang, 2015; Li and Zhang, 2019). In the existing studies, some researchers proved the existence of psychic income through measuring with relevant scales, and further predicted that psychic income might affect residents’ attitudes toward the sports event (Wilson et al., 2000; Hastie, 2001; Oja et al., 2018). In some other literature, researchers deemed involvement an important variable in sports psychology, which could explain and affect behavior and attitude (Liu et al., 2014; Zhang, 2015; Pan et al., 2018). Sport event involvement refers to the host city residents’ awareness and knowledge of it, and the extent of their enthusiasm, interest, and participation in the event (Bee and Havitz, 2010; Liu et al., 2014). The sport event involvement might be an important influencing factor of the relation between the residents’ psychic income and attitudes (Crompton, 2004; Gibson et al., 2014; Liu, 2017; Ishac et al., 2018; Kim et al., 2019), but there is a lack of empirical evidence to justify it, especially in the context of China. Therefore, it requires more work in studying how the residents’ sports event involvement affects their psychic income and supportive attitudes.

Using the Shanghai Masters tennis tournament as an example, this study seeks to address this gap by exploring the causal effect between local residents’ psychic income and attitudes, in which sports event involvement works as a moderating factor. Shanghai Masters is the only ATP 1,000 event in the area of Asia, it is the tennis tournament with the longest history (over 20 years) in China, which is well-known among the local residents. The local government keeps developing it into a major brand of sports events. As Shanghai Master is one of the three most influential sport events in Shanghai in terms of public attention, profession, and contribution, it is representative to use Shanghai Master as the case to study the relation among residents’ sport event involvement, psychic income, and supportive attitudes of all the similar kinds of sport events.

## Literature Review

### Psychic Income and Its Measurement

Psychic income was originally used in the field of human resources management to outline the sense of emotional satisfaction or psychic pleasure to an individual, that which cannot be classified or shown as actual material benefits (Brent Ritchie, 1984). Crompton described the psychic income of the residents from the host area as emotional and psychic benefits, even if they did not personally participate in sports events (Crompton, 2004). In his concept, Crompton proposed a seven-dimension psychic income paradigm: (1) community pride resulting from increased visibility, it refers to the residents’ sense of self-respect due to increased visibility nationally and internationally owing to an event (Gibson et al., 2008). Several scholars refer to this impact as the “showcase effect” of media coverage (Horne and Manzenreiter, 2004; Kim and Walker, 2012) since it is the media coverage that improves the visibility of the community, which might possibly improve the profile and investment opportunities of the region. For instance, during the period of hosting the sports event, the media of different sources would report the event, not only limited to the game, but also cover the community details, the performance of management operation, the community culture, and even the civic attributes. This revelation would bring a sense of self-respect to the residents of the community, which is regarded as a kind of long-term benefit (Fredline and Faulkner, 2000; Kim and Walker, 2012); (2) civic pride from being a sports event host city for sports events, it refers to the pride from the improvement and praise of the city’s image, status, and ability as a sports event host city (Fredline and Faulkner, 2000). The success of a sport event can be translated into a positive “can-do” attitude (Crompton, 2004), which could then improve the community residents’ satisfaction and pride (Mules, 1993), and communicate a kind of positive belief to the residents, make them believe their community is capable of dealing with important tasks (Kim and Walker, 2012); (3) pride in efforts to revive deteriorated areas, sport events are often part of a city’s regeneration strategy designed to improve the city’s profile, they serve as urban redevelopment catalysts (Chapin, 2004). The hosting of sport events can revitalize deteriorated community areas, especially aging downtown ones (Hall, 1992; Austrian and Rosentraub, 2002; Teye et al., 2002; Chapin, 2004). Proponents of revitalization have argued that new and renovated infrastructures give a competitive advantage to the community, and provide opportunities for hosting future events. Some studies suggest that urban renewal, old area reconstruction, and community vitality restoration through hosting sports events would increase the community awareness and pride of the residents (Kim and Walker, 2012); (4) increased collective self-esteem, refers to the perception of the resident himself/herself as a member of the community that is hosting the sports event, and the assessment of the value and affective importance of being a member of this community (Eckstein and Delaney, 2002). As the current society is highly complex, members are likely to be alienated from each other. However, sport is a medium through which cities and residents express their identity and shared beliefs (Branscombe and Wann, 1991; Heere and James, 2007). Therefore, hosting sports events can enhance community self-esteem, including improved quality of life (Delamere, 2001; Ko and Stewart, 2002; Haley et al., 2005). (5) With tangible focus on social bonding, it specifies that sports events increase the interactions of the local residents including friendships, sentiment, and social participation (Wann, 1995; Trail and James, 2001; Funk et al., 2002). Social interaction is regarded as an important factor for individual well-being in a society. Several researchers have supported the idea that sport can increase affiliations among community members (Washington and Karen, 2001; Collins, 2004; Crompton, 2004). Watching games with families increases family ties (Wann, 1995; Funk et al., 2001; Eitzen, 2015); talking about sports events with others (Moore, 2015), or participating in event-related social activities, ancillary events, theme activities, individual’s interaction, bonding, and communication with others would be increased (Chalip, 2006). Although events are temporary and their duration is fleeting, host community members have various tangible socializing opportunities pre- and post-event (Eitzen, 2015). The enhanced social bonding improves social relations, generates teamwork, and decreases feelings of social alienation (Frey and Eitzen, 1991; Washington and Karen, 2001; Collins, 2004; Crompton, 2004); (6) excitement from the events and visitors refers to the residents’ emotionally stimulated state from hosting a sports event, a result of both the event itself and the influx of visitors to the local community. The enhanced excitement among community members and visitors is mutually transferable and contagious (Christine Green, 2001; Chalip, 2006). The local government can make good use of the sports event to provide particular experiences for residents and tourists (Devine and Devine, 2004), like the culture or entertainment celebrations, carnivals, sponsors’ promotion activities, etc. (Kidd, 1992). Sometimes the games of the sports events are more exciting, which can stimulate people (Gibson et al., 2014). Gibson and Hardman (1998) explained that excitements were mimetic experiences that generated emotion, specific to the individual’s positive perception of the event and elevated consumption behavior (Kim and Walker, 2012). Therefore, the elevation of the residents’ emotion during the event is deemed an extra psychic income (Kim and Walker, 2012); and (7) emotional involvement with a sports event, refers to the residents’ increased sense of motivation, arousal, or interest toward hosting sports events (Havitz and Dimanche, 1999). Some researchers argue that sport activity would improve an individual’s community satisfaction if he or she has high involvement in sports events. Havitz and Dimanche (1999) claimed that individuals’ event emotional involvement occurred when they developed arousal or interest in participating in the event. Emotional involvement is helpful to promote community residents’ awareness of the event’s significance, interest, and enjoyment (Hall, 1992; Kim and Walker, 2012).

Kim and Walker (2012) developed a multidimensional Scale for psychic income (SPI), which was grounded in the seven dimension psychic income paradigm of Crompton (2004). The SPI includes 5 dimensions: (1) community pride as result of enhanced image, it refers to the residents’ pride and satisfaction resulting from the improvement of the city’s visibility and image due to the increasing media coverage of the sport event; (2) enhanced community attachment, it refers to the hosting of the sport event can enhance the sense of community belonging and social cohesion of the residents, facilitate more social interaction and communication, develop harmonious social relations, and bring in euphoria; (3) event excitement, it refers to the festive atmosphere brought by the sport event, which then brings euphoria to the residents; (4) community excitement, it refers to the excitement brought by the derivative activities rather than the focal event itself, like the activities of sponsorship, training, and promotion; (5) pride in efforts to improve community infrastructure, it refers to the satisfaction and pride resulting from the improvement of city (re)construction and public service through hosting the sport event. Through the empirical test of the residents of the Super Bowl XLIII host city, the results prove the SPI can measure the residents’ psychic income; Tampa Bay residents have received significant psychic income from hosting Super Bowl XLIII.

Following the above research, some other researchers have done some empirical researches about psychic income for sports events of different scales or types. For instance, Gibson et al. (2014) adopted a four-dimension scale of psychic income: (1) increasing the community spirit and pride; (2) enhancing the national pride and patriotism; (3) making residents satisfied with themselves and their community; (4) making people get together for celebrations. In this study of the impact of the 2010 FIFA World Cup, the results suggest psychic income increased among South African residents 8 months after the event. Oja et al. (2018) used a five-dimension scale: (1) city excitement; (2) improvement of infrastructure; (3) city pride; (4) city attachment; (5) event excitement. He measured the residents’ psychic income before and after the 2012 Major League Baseball All-Star Game and the results suggest that some components of psychic income dissipated after the event. Ishac et al. (2018) employed a five-dimension scale to study the young residents’ psychic income from Handball World Championship 2015: (1) Community pride as a result of enhanced image; (2) enhanced community attachment; (3) event excitement; (4) community excitement; (5) Pride in efforts to improve community infrastructure. Weight et al. (2019) adopted a seven-dimension scale: (1) community pride resulting from increased visibility; (2) civic pride from being a major college athletics host city; (3) pride in efforts to resuscitate deteriorated areas; (4) enhanced collective self-esteem; (5) tangible focus for social bonding; (6) excitement from athletic events and visitors; (7) emotional involvement with a college athletic department. This study examined the psychic income the residents of Chapel Hill, North Carolina receive from the university athletics department. The results suggest that although the psychic income and its relevant scales were developed for mega sports events, they could be available to measure the psychological impact of small-scale sports events on the residents of the host community.

In the domestic researches, Liu (2017) examined the psychic income of local residents from the 2008 Beijing Olympics from the perspective of Olympic legacy in the context of China. By considering the different features between the Olympics and Super Bowl, Liu revised Kim and Walker’s (2012) SPI into a new scale with seven dimensions: (1) pride in the enhanced international image; (2) enhanced community attachment and social bonding; (3) event excitement; (4) pride in improved infrastructure; (5) pride in being an Olympic host city; (6) pride in the national team performance; (7) increased culture confidence, which was used to measure the local residents’ psychic income from the 2008 Beijing Olympics. The results suggest that the local residents’ psychic income can last for years after the 2008 Beijing Olympics, which justify psychic income as an important cultural legacy of sports events. Liu suggested that host cities of mega-sporting events and events owners should pay special attention to intangible benefits including psychic income rather than just focusing on economic benefits to maximize the positive impact of hosting these events. He also suggested that SPI might be available for other types of domestic sports events, which could serve as a reliable and valid assessment instrument for both academics and events owners.

### Psychic Income and Host Community’s Attitude

In psychology, an attitude refers to “an individual’s overall evaluation of an object, issue, or person,” which means the degree to which an individual views an object favorably or positively (Koo and Lee, 2019). Based on the extant literature, the attitudes of the residents toward sports events are affected by various factors such as residents’ perception, public involvement, residents’ psychic emotions, residents’ preference for events, sense of community belonging, community attachment, and residents’ demographic sociological parameters (Huang, 2015; Lian and Huang, 2017; Liu, 2017; Han et al., 2018). Some authors argued that the psychic income of the event is an important content of the subjective feelings of the residents in the host area, and also an important factor affecting the attitude toward the sports event (Lian and Huang, 2017; Liu, 2017; Oja et al., 2018; Weight et al., 2019).

According to social exchange theory, any exchange is a kind of reciprocal behavior, and the residents’ support for the event is the result of their comprehensive evaluation of the impact of the event. The beneficial evaluation results would lead to positive attitudes and support intentions. The psychic income perceived by residents, i.e., the emotional gain, is a beneficial evaluation result and a positive emotional response reflecting “comprehensive evaluation and feeling” toward the sports event. It is believed that psychic income can effectively predict the residents’ support for the event (Liu, 2017; Oja et al., 2018).

Furthermore, the Emotion Appraisal Theory suggests that individual emotions are formed based on one’s reaction to outward things or settings. Individual emotions are the outcome of an individual’s perception, cognition, and appraisal of things and settings. The positive emotions can boost an individual’s initiative, as well as his or her positive behavioral intentions (Li et al., 2019; Zhou, 2019).

The Broaden-and-Build Theory of Positive Emotions supports that emotions are the product of the interaction between an individual and the outside environment. Emotions are the judgments formed through the perception of the outside stimulus and self-adjustment (Hu and Li, 2019). Compared with mood, emotions are more consistent, profound, and stable. This theory suggests that positive emotions can strengthen and expand an individual’s instruction system, leaving people in a state of actively acquiring information and in-depth thinking, and further specifically impacting the behaviors. Positive emotions can positively affect an individual’s cognition and behavior intention, guide him or her to get information, think, and respond actively. Moreover, the expansion-construction function of the positive emotion to an individual’s thoughts and behavior is persistent and spiral, which brings a long-term effect to an individual (Bao, 2018).

To understand the concept of psychic income from the perspective of emotions: psychic income includes both the short-term emotions like excitement, and the long-term emotions such as pleasure, pride, and satisfaction. In light of the Emotion Appraisal Theory and the Broaden-and-Build Theory of Positive Emotions mentioned above, psychic income, as a kind of residents’ psychological emotion and emotional reaction by appraising the sports event, supplements and explains the evaluation mechanism of emotions and behavioral intentions. To be more specific, psychic income represents a kind of positive and optimistic mood and emotion, as well as a positive and helpful appraisal outcome, which may bring a positive impact on attitude and behavior. The Broaden-and-Build Theory of Positive Emotions does not only emphasize that psychic income positively affects behavioral intentions, but also highlights the significance of psychic income of identifying, guiding, cultivating, and utilizing the positive emotions, which results in sports events’ sound, balanced, sustainable, and high-quality development.

These are the rationale for the first hypothesis of the study:

H1: The psychic income of a sport event has a positive impact on the residents’ attitude toward the event.

### Involvement

Involvement refers to the person’s perceived relevance of objects based on their own internal needs, values, and interests; it is the individual’s degree of devotion to an object, an action, or an activity, as well as the production of enthusiasm and interest (Zaichkowsky, 1985). Residents’ involvement in sports events is based on the local residents’ understanding and knowledge of sports events, their enthusiasm, interest, and involvement in hosting sports events (Stevens and Rosenberger, 2012). The content of the event involvement contains value, interest, information, and other perceptions (Zaichkowsky, 1994).

In Crompton’s (2004) original description of psychic income, resident involvement was described as one of the seven dimensions of psychic income, but involvement was not retained as a dimension to measure psychic income in the study by Kim and Walker (2012). It is suggested that residents’ involvement in events may be an important factor affecting psychic income and attitudes (Stevens and Rosenberger, 2012; Liu, 2017; Weight et al., 2019), but the is a lack of empirical research to understand mechanisms at work in the existing literature.

In the field of consumption, sponsorship, leisure, tourism, and sports event, involvement is considered a key variable, and scholars generally agree that involvement is a useful concept for explaining behavior and attitudes and is an important moderator of consumer attitudes (Pan et al., 2018; Vera and Espinosa, 2019). Some scholars think it is an important moderator of consumer attitudes, decisions, and behaviors (Johnson et al., 2001). For instance, Cadogan et al. (2009) suggested involvement could moderate the relation between purchase attitude and purchase frequency; Jung and Tey (2010) found consumers with high involvement had a better evaluation for the augmented products with medium fit than products with a high or low fit, whereas, in the situation of low involvement, consumer’s evaluation of the augmented products was positively correlated with the perceived fit (Li et al., 2015). The findings justify consumer involvement has a significant moderate effect between perceived fit and brand extension evaluation. Li (2019) argued the product involvement had a reverse moderate effect on the mechanism of national brand image’s impact on consumer’s willingness to buy: high product involvement would weaken the impact of national brand image on willingness to buy, whereas low involvement would intensify the impact of national brand image on willingness to buy.

Liu et al. (2009) argued tourists with high accommodation products would pay more attention to product information and know more about the connotation of ecotourism, the high involvement would lead to tourist’s decision of selecting ecotourism products; tourists with higher involvement would hold more positive opinions about the relations among ecotourism product and their interest, target, and value. As a result, product loyalty would be formed, which would enhance consumers’ environmental intention. Yuan et al. (2019) argued that there was an underestimation of tourism involvement as a predictor for residents’ perceived tourism impacts and their support for tourism. Her findings showed that residents’ support for tourism was the result of a complete behavior generation process, which had gradually formed through tourism involvement, cognition, affection, and behavior intention. Residents’ tourism involvement does matter to predict the attitudes and understand the behaviors (Poiesz and de Bont Cees, 1995; Balduck et al., 2011), the tourism involvement might affect residents’ communication process with the development of tourism, then finally affect their attitudes (perception of tourism’s impact) and behaviors (support for the tourism). The results highlight the importance of involvement and emotional attitudes in determining residents’ tourism attitudes.

Regarding the studies of involvement and sports event, Kim et al. (2019) discussed how would sports involvement affect the residents’ perception of the event, quality of life, and their supportive attitudes to the 2018 Pyeongchang Winter Olympics from the perspective of the host city residents. The author agreed that sports involvement had a positive effect on the perception of the event’s impact and quality of life, then had a significant impact on the early support of the Olympic Games. High involvement in sports may help residents to establish a positive link between sports activities and community member attitudes (Balduck et al., 2011). The residents with high involvement might be willing to accept the positive information of the event and weaken the negative information (Ap, 1992). As a result, a more positive supportive attitude can be formed (Gelan, 2003). Similarly, Armenakyan et al. (2017) argued that involvement in sports events would affect individuals’ beliefs, expectations, and evaluation of the sports event. Individuals closely related to sports events tend to maintain a positive impression of it because they tend to think that sports events are related to themselves, and can meet their own needs, values, and interests (Pan et al., 2018). Regarding the studies of the image of host cities, Kim and Kaplanidou (2019) agreed residents with higher event involvement tended to have a positive impression of the host country, the reason was that individuals with higher involvement usually paid more attention to the media coverage, and had stronger emotional investment, which made the image of the event more positive, and efficiently transferred to the image of the host country. The study highlighted in the pre-event evaluation of the factors that influence the image of the host country, there was no other influence path except the event involvement, which indicated the importance of event involvement to the image of the host country. Therefore, hypothesis 2 is put forward:

H2: Sports involvement has a moderating effect between psychic income and attitude.

**FIGURE 1:**
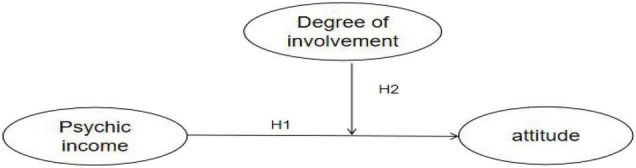
The conceptual model framework of psychic income, involvement, and support attitude.

The theoretical model consisting of the two hypotheses is shown in Figure 1.

## Research Methods

### Participants

Data were collected from residents of Shanghai (*N* = 1,302), who had lived in Shanghai for over 1 year, and were aware of the sports event in the survey. Of the 1,302 respondents, 751 (57.7%) were males and 551 (42.3%) were females. Among the respondent, 31.9% were aged between 18 and 28, followed by 36.1% between 29 and 40, 17.6% between 41 and 55, with the remainder 21% aged above 56 years old (see Table 1).

**TABLE 1:** Demographics of samples.

Variables	Items	Qty	Ratio (%)	Variables	Items	Qty	Ration (%)
Gender	Male	751	57.68	Relation to the even	Spectator	663	50.92
	Female	551	42.32		Others	639	49.08
Age	18 ∼28	415	31.87	Employment	Employed	757	58.14
	29∼40	470	36.10		Unemployed	306	23.50
	>40	417	32.03		Student	239	18.36
Income (CNY)	≤6,999	736	56.53	Frequency of watching games	0	513	39.40
	7,000∼14,999	375	28.80		1∼2	586	45.00
	>15,000	191	14.67		>3	203	15.60
Education Level	Junior high school or higher vocational school	289	22.20	Years of dwelling	1∼5	420	32.26
	Junior college or bachelor	832	63.90		6∼15	349	26.80
	Master or above	181	13.90		>15	533	40.94

### Instruments

All the variables of the measurement came from the existing scales and were modified according to the current study. As part of the items came from the scales developed by overseas scholars, the translation of Chinese-English and rewording were conducted to formulate the preliminary scale. A focus group interview was also adopted to assess the content validity, research hypothesis, and wording of the questionnaire. Furthermore, the scale was sent to a panel consisting of ten professors to improve its content validity and integrity. After the above steps, the final questionnaire was formulated with four major parts: residents demographics, psychic income, sport event involvement, and supportive attitude.

The psychic income measurement is adapted from the SPI measurement scale of Crompton (2004), Kim and Walker (2012), and Liu (2017), it has been modified according to the features of Shanghai Master. The measurement contains five dimensions including pride resulting from improved international image, emotional satisfaction resulting from enhanced social belonging and interaction, good feeling derived from competition passion, good feeling from the city passion, pride resulting from the urban development and infrastructure improvement, and 26 items in total (see Table 2).

**TABLE 2:** Descriptive statistics of sample data.

Measurement dimension and measurement item	*M*	*SD*	Kurtosis	Skewness
**1 Degree of involvement**				
1. It’s important for me to follow the tournament	3.32	0.937	0.099	–0.163
2. Following the event is one of my main ways of leisure	3.28	1.013	–0.396	–0.184
3. I am very concerned about the relevant information of the event	3.31	0.989	–0.157	–0.185
4. It is worthwhile for me to spend time learning about the event	3.35	1.003	–0.322	–0.241
5. My interest in the tournament reflects the nature of my interests	3.49	1.010	–0.093	–0.439
6. My interest in the tournament reflects my values	3.37	1.059	–0.349	–0.294
**2 Psychic income**				
**2.1 Pride brought about by improved international image**				
1. I am proud that the event has demonstrated Shanghai’s ability to host important international events	3.92	0.897	0.478	–0.704
2. I am proud that the event has shown the world a positive image of Shanghai	4.01	0.870	0.211	–0.673
3. I am proud that the event has gained more (international) recognition for Shanghai	4.04	0.888	0.417	–0.784
4. I am proud that the event shows that Shanghai has the ability to host other (important international) events	4.04	0.888	0.570	–0.824
5. I am proud that the event has given Shanghai more international exposure	4.02	0.902	0.627	–0.812
**2.2 Social belonging and enhanced social interaction lead to emotional satisfaction**				
6. The event has increased my social contact with others, which makes me feel satisfied	3.50	0.902	0.125	–0.410
7. The event provided me with an opportunity to show myself, which made me feel satisfied	3.40	0.948	–0.194	–0.231
8. The event has earned me the respect of others, which made me feel satisfied	3.46	0.981	–0.258	–0.269
9. The event has increased my contact with social groups, which makes me feel satisfied	3.53	0.944	–0.189	–0.302
10. The event has improved my sense of self, which makes me feel satisfied	3.53	0.946	–0.133	–0.317
11. The tournament has increased my friendship with others, which makes me feel satisfied	3.59	0.930	–0.053	–0.427
12. Holding the event has increased my connection with my family, which makes me feel satisfied	3.48	1.004	–0.367	–0.319
13. The event has created more opportunities for me to meet new friends, which makes me feel satisfied	3.69	0.956	–0.069	–0.477
**2.3 Competition passion brings good feeling**				
14. I enjoy watching the Games	3.72	0.842	0.314	–0.498
15. The event added a lot of joy to me	3.74	0.875	–0.047	–0.404
16. I have good memories of the event	3.77	0.899	–0.130	–0.424
17. I am very excited about the event	3.68	0.897	–0.180	–0.332
18. The event increased my interest in the game	3.78	0.887	0.218	–0.552
**2.4 City passion brings good feeling**				
20. The event has brought new excitement to the city, which makes me feel good	3.87	0.790	0.498	–0.477
21. The event will increase the city’s cultural and sports activities, which makes me feel good	3.92	0.784	0.348	–0.496
22. The event adds to the entertainment of the city, which makes me feel good	3.95	0.815	0.564	–0.645
23. The event has enriched my spiritual and cultural life, which makes me feel good	3.95	0.819	0.377	–0.593
**2.5 Urban development and infrastructure improvement bring pride**				
24. The games have improved public infrastructure, which makes me feel proud	3.86	0.853	0.487	–0.593
25. The event has improved the quality of public services, which makes me feel proud	3.84	0.844	0.038	–0.458
26. The hosting of the event has increased the opportunities for the development of related industries and created more jobs, which makes me feel proud	3.90	0.875	–0.149	–0.477
**3 Supportive attitude**				
1. I hope that shanghai will continue to host the event in the future	4.13	0.821	0.659	–0.793
2. I hope shanghai will hold more similar sports events	4.17	0.817	0.687	–0.866
3. I hope to have more opportunities to participate in this event (directly participate, watch the event, participate in other related activities)	3.99	0.861	–0.272	–0.497
4. I am willing to contribute to this event (volunteers, publicity and recommendation, self-civilized restraint, etc.)	3.92	0.868	0.106	–0.552
5. I agree that the government should use public funds to provide certain government subsidies for Shanghai to host this event	3.91	1.005	0.189	–0.771

The involvement scale is adopted from the involvement consumer involvement profile (CIP) scale, the personal involvement inventory (PII) scale (Laurent and Kapferer, 1985; Zaichkowsky, 1994), which outlines 6 items including importance, value, interest, and information. Attitude measurement is adopted from Huang et al. (2016) and Lian and Huang (2017), and contains 5 items such as “hope to continue to host the event,” etc. (see Table 2 for specific items).

All the measurement is done through five-point Likert scale items, anchored from strongly disagree (1) to strongly agree (5).

### Procedures

Following the survey packet, the test administration was accomplished through individualized, face-to-face interviews that were conducted by trained investigators. The survey was conducted in September 2019, 1 month prior to Shanghai Master. On-site completion and collection of the questionnaires were conducted. Respondents were intercepted and a filter question was used to identify if they were aware of Shanghai Master and had lived in Shanghai for over 1 year. Respondents who met the above two conditions were invited to complete the questionnaires. At the beginning of an interview, honest responses were sincerely sought; in the meantime, confidentiality and anonymity were assured. To improve the data authenticity as well as the participation of the respondents, a small souvenir would be offered if the respondent completed the questionnaire with validity.

According to the administrative division, Shanghai is divided into 16 districts/counties, each has a population ranging from 700 thousand to 5 million. The sampling size is determined by selecting one out of 10 thousand residents of each district. In fact, multi-level stratified sampling is adopted in this study. The survey was conducted on both weekdays and weekends, in public places with a dense population (e.g., streets, stadiums, entrances/exits of subways). In total 1,500 questionnaires were distributed, and 1,405 were collected, some of them were dropped (significant missing data, wrong data, totally same data), resulting in 1,302 (86.8%) valid questionnaires retained for further analysis.

### Statistical Methods

In this study, SPSS 25.0 (IBM, New York, United States) was used to conduct an analysis of descriptive statistics, reliability, and validity; AMOS 24.0 (IBM, New York, United States) was used to accomplish confirmatory factor analysis; the macroprogram Model 1 of SPSS 25.0 was used to test the moderating effect.

## Results

The results showed that the respondents elicited psychic income in all the measurement dimensions including pride. The mean value in Table 2 suggests the dimensions of the highest to lowest psychic income perceived by the respondents as follow: pride resulting from an improved international image, good feeling from the city passion, pride resulting from the urban development and infrastructure improvement, good feeling derived from competition passion, emotional satisfaction resulting from enhanced social belonging and interaction. Even the dimension with the lowest mean score is above 3.5. According to the result of the Likert scale, the five dimensions of the respondents’ perceived psychic income were positive. The mean scores of the sports event involvement and supportive attitude are 3.35 and 4.03, respectively. Overall, the respondents of Shanghai hold positive supportive attitudes toward hosting Shanghai Master.

To assess the reliability of the factors identified in the scale, the following three tests were conducted: Cronbach’s alpha (α), construct reliability (CR), and averaged variance extracted (AVE). Cronbach’s alpha (α) coefficients (i.e., internal consistency values) indicate the relationship among items. CR is an internal consistency measure that accounts for measurement errors of all indicators (Bagozzi et al., 1981). The internal consistency (α) value and CR are suggested to be equal to or greater than the 0.70 cut-off point (Bagozzi et al., 1981; Nunnally and Bernstein, 2010). The AVE values assessed the variance captured by the indicators relative to measurement error. An AVE value above 0.50 is considered acceptable (Bagozzi et al., 1981). The results showed the AVE of both Competition passion brings good feeling and City passion brings good feeling dimensions are below 0.5. After further examination, the item Participating in competitions or related activities has given me a sense of Pride (e.g., carnivals, camps, challenges, shows, etc.) was supposed to be deleted due to its low normalized factor load (<0.50). According to the results of CFA after the deletion of the item (see Table 3), the new questionnaire has good reliability and convergence validity.

**TABLE 3:** Analysis of reliability and convergence validity.

Measurement dimension and measurement item	Normalized factor load	CR	AVE	Cronbach’s α
**1 Degree of involvement**		0.932	0.697	0.913
1. It’s important for me to follow the tournament	0.805			
2. Following the event is one of my main ways of leisure	0.841			
3. I am very concerned about the relevant information of the event	0.856			
4. It is worthwhile for me to spend time learning about the event	0.859			
5. My interest in the tournament reflects the nature of my interests	0.822			
6. My interest in the tournament reflects my values	0.825			
**2 Psychic income**				
**2.1 pride brought about by improved international image**		0.917	0.687	0.916
1. I am proud that the event has demonstrated Shanghai’s ability to host important international events	0.741			
2. I am proud that the event has shown the world a positive image of Shanghai	0.733			
3. I am proud that the event has gained more (international) recognition for Shanghai	0.755			
4. I am proud that the event shows that Shanghai has the ability to host other (important international) events	0.718			
5. I am proud that the event has given Shanghai more international exposure	0.736			
**2.2 Social belonging and enhanced social interaction lead to emotional satisfaction**		0.926	0.611	0.926
6. The event has increased my social contact with others, which makes me feel satisfied	0.683			
7. The event provided me with an opportunity to show myself, which made me feel satisfied	0.754			
8. The event has earned me the respect of others, which made me feel satisfied	0.778			
9. The event has increased my contact with social groups, which makes me feel satisfied	0.765			
10. The event has improved my sense of self, which makes me feel satisfied	0.769			
11. The tournament has increased my friendship with others, which makes me feel satisfied	0.728			
12. Holding the event has increased my connection with my family, which makes me feel satisfied	0.730			
13. The event has created more opportunities for me to meet new friends, which makes me feel satisfied	0.738			
**2.3 Competition passion brings good feeling**		0.893	0.626	0.892
14. I enjoy watching the Games	0.639			
15. The event added a lot of joy to me	0.734			
16. I have good memories of the event	0.734			
17. I am very excited about the event	0.701			
18. The event increased my interest in the game	0.671			
**2.4 City passion brings good feeling**		0.845	0.576	0.844
20. The event has brought new excitement to the city, which makes me feel good	0.606			
21. The event will increase the city’s cultural and sports activities, which makes me feel good	0.616			
22. The event adds to the entertainment of the city, which makes me feel good	0.604			
23. The event has enriched my spiritual and cultural life, which makes me feel good	0.608			
**2.5 Urban development and infrastructure improvement bring pride**		0.799	0.570	0.793
25. The Games have improved public infrastructure, which makes me feel proud	0.672			
25. The event has improved the quality of public services, which makes me feel proud	0.668			
26. The hosting of the event has increased the opportunities for the development of related industries and created more jobs, which makes me feel proud	0.596			
**3 supportive attitude**		0.890	0.618	0.842
1. I hope that shanghai will continue to host the event in the future	0.804			
2. I hope shanghai will hold more similar sports events	0.820			
3. I hope to have more opportunities to participate in this event (directly participate, watch the event, participate in other related activities)	0.780			
4. I am willing to contribute to this event (volunteers, publicity and recommendation, self-civilized restraint, etc.)	0.778			
5. I agree that the government should use public funds to provide certain government subsidies for Shanghai to host this event	0.747			

Discriminant validity is the extent to which a factor is distinct from other factors. Kline (2016) suggested that discriminant validity could be established when inter-factor correlation was below 0.85. In this current study, inter-factor correlations ranged from 0.390 (between Pride Brought About by Improved International Image and Social belonging and enhanced social interaction lead to emotional satisfaction) to 0.762 (between City Passion Brings Good Feeling and Urban Development and Infrastructure Improvement Bring Pride). Another way to evaluate discriminant validity is comparing the square root of AVE with the correlation coefficients among the factors. It is recommended that the square root of the AVE of a factor should be greater than the correlations between one factor and any other factor in the model (Bagozzi et al., 1981; Hu and Bentler, 1999). Findings in Table 4, notwithstanding the correlation between City Passion, Brings Good Feeling (2.4) and Urban Development and Infrastructure Improvement Bring Pride (2.5) presents a value (*r* = 0.762) higher than the square root of AVE (0.759), the approximation of the two values suggested a satisfying discriminant validity of the scale.

**TABLE 4:** The square root and average variance extracted (AVE) correlation matrix of the psychological income.

Factor	1	2	3	4	5
1. Pride brought about by improved international image	0.829				
2. Social belonging and enhanced social interaction lead to emotional satisfaction	0.390**	0.782			
3. Competition passion brings good feeling	0.490**	0.753**	0.791		
4. City passion brings good feeling	0.631**	0.581**	0.698**	0.759	
5. Urban development and infrastructure improvement bring pride	0.538**	0.512**	0.538**	0.762**	0.755

**Refers to P < 0.01(the same as below).

In order to reduce the common method bias, anonymous responses were used in the survey, and the Harman single factor test was used. The results showed that the characteristic root values of 7 factors were greater than 1, and the explained variation rate of the first factor was 39.811%, which was lower than the 40% critical value, the common method deviation of data measurement is acceptable.

To test the goodness-of-fit of the model, multiple indices like Chi-square degree of freedom ratio (CMIN), comparative fit index (CFI), Tucker-Lewis Index (TLI), and root mean square error of approximation (RMSEA) were used in this study. According to Hu and Bentler’s (1999) criterion, the model fits the data well when χ^2^/df is below 3, the model is acceptable when χ^2^/df is between 3 and 5, while the fit is poor when χ^2^/df exceeds 5. CFI and TLI would be better if they are above 0.9 and close to 1. RMSEA should be below 0.06 (Hu and Bentler, 1999). In this study, the hypothesized model’s fit was examined at first, the results showed χ^2^/df = 4.603, *P* < 0.05 (P is determined by the sample size, the significance of chi-square criterion is: when *N* ≤ 150, *P* = 0.01; *N* = 200, *P* = 0.001; *N* = 250, *P* = 0.0005, *N* ≥ 500, *P* = 0.0001; while when *N* ≥ 1,000, *P* = 0.0001 is still not low enough, which may lead to the rejection of the model even it is with large chi-square and good fit); therefore, other indices are needed for an overall assessment. In this study, CFI = 0.955, TLI = 0.949, RMSEA = 0.053, which suggested the model fit the data well.

**FIGURE 2:**
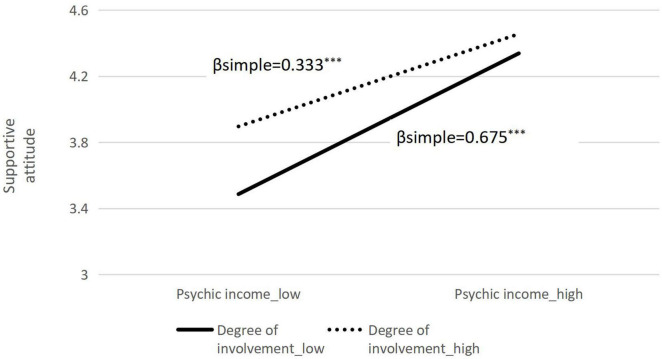
Moderating effect of involvement between psychic income and attitude. ***Refers to *P* < 0.001 (the same as below).

The hypotheses were tested through the macroprogram Model 1 of SPSS 25.0. The average values of psychic income, involvement, and support attitude are used. The results are shown in Table 5. Psychic income has a significant positive effect on attitude (β = 0.963, *p* < 0.05), and the product of involvement and psychic income has a significant positive effect on attitude (β = –0.083, *p* < 0.05). A further simple effect analysis showed that for low-involved residents (M–1 *SD*), the positive predictive effect of psychic income on supportive attitudes was significant (β simple = 0.675, SE = 0.051, 95% CI = 0.573–0.776); involved residents (M + 1 *SD*), psychic income has a significant positive predictive effect on support attitudes (β simple = 0.333, SE = 0.060, 95% CI = 0.214–0.453; β simple is reduced from 0.675 to 333) (see Figure 2 for details). This shows that, compared with low-involved residents, the psychic income of high-involved residents has a lower impact on the support attitude. In addition, the data show that the spectators’ involvement (*M* = 3.6060, *SD* = 0.7702) is significantly higher than that of non-spectators’ (*M* = 3.0875, *SD* = 0.8216), *P* < 0.001, which to some extent indicates that there are differences in the relationship between the spectators and non-spectators in psychological benefits and attitudes.

**TABLE 5:** Moderating effect test.

Independent variable	Dependent variable	
	Attitude	Attitude
Psychic income	0.580***	0.963***
Involvement		0.724***
Involvement × psychic income	–	–0.141***
R2	0.371	0.415
F	725.83***	290.62***

***Refers to P < 0.001.

## Discussion

From the perspective of the local residents, this article drew on relevant discussions in the fields of consumer science, sociology, and psychology to study the relationship among the involvement, psychic income, and support of major sports events.

The results of the study confirmed that psychic income would play a positive role in event support attitudes, which is in line with existing literature (Walker et al., 2013; Gibson et al., 2014; Liu, 2017; Thomson et al., 2020). More specifically, the higher the psychic income received by the residents of the hosting site in the event, the more likely they are to develop supportive attitudes. This is consistent with the social exchange theory where good feelings tend to stimulate positive exchanges, prompting positive attitudes toward the event and generating support. These supportive attitudes include the expectation that Shanghai will continue to host this event, and more similar sports events in the future, and the expectation that Shanghai can have more opportunities to participate in and the willingness to contribute to the event, and support the government to use public funds and provide subsidies. This is consistent with the hypothesis proposed by scholars at home and abroad, that is, stronger positive psychic income leads to stronger supportive attitudes; the positive impact of perception on intention is significant (Brent Ritchie, 1984; Liu, 2017). This study also extends Crompton’s (2004) expectation of providing support and quantitative theoretical foundation for the government’s hosting of mega sports events in the domestic context.

The findings suggested that (1) sponsors and owners of the event should attach great importance to residents’ psychic income from the event. The event is an all-around exhibition of the city’s history, culture, and features. It is an opportunity to extend the understanding of the city and realize the identification with the city for both external tourists and local residents. The event also promotes the infrastructure construction, improvement of public service system, and capacity as well as the promotion of the upstream, downstream, and derivative industries of the event, which is one of the ways for brand events to improve the city’s integrated development and residents’ benefits. Through the active guidance and effective cultivation of all parties in the event, the residents’ psychic income and benign emotions in the high-quality competition can be reasonably optimized. In particular, extensive publicity of the image and infrastructure improvement, the development of supportive industries of the city, and other positive externalities relating to the event will inspire and enhance the public’s pride in hosting the sports event. Effective links must be formed among work for the benefits of the city, the event, and the citizens so that the public sense of gain can be enhanced. (2) innovation, development, and enrichment of the event dimension and derivative activities help maintain the novelty and attraction of brand events, create a virtuous circle of urban event atmosphere, so as to enhance the public’s “passion for the event,” “passion for the city,” and “social belonging and interaction.” It is even more necessary to deeply stimulate the public through sports cultivation and event cultural precipitation, inspiring residents to experience, fit in with, recognize and love the unique charm of the event, then resonate with the value identification of the event, eventually gain a happy and satisfying psychological feeling.

This study also confirmed the positive moderating effect of involvement between psychic income and supportive attitude. Scholars found that residents would have different event perceptions, decisions (Laurent and Kapferer, 1985; Yuan et al., 2019; Safshekan et al., 2020), and attitudes along with the varying involvement. Involvement can be deemed a moderating factor of an individual’s attitude (Chen and Funk, 2010). The results were largely in line with previous research, as Song et al. (2014) found empirically that the higher the consumer’s brand involvement, the stronger the role of their experience with that brand on their propensity to consume. Armenakyan et al. (2017) also found that involvement in sporting events affects one’s beliefs, expectations, and evaluations of sporting events.

In this study, residents with a high degree of involvement are more interested in the event and pay more attention to it. With more information about the event, it is easier to appreciate the pride that the event brings to Shanghai’s international image and to recognize that the successful hosting of the event exemplified the city’s management and governance capability. It is also easier to perceive the improvement of the city’s infrastructure, public services, and industrial development opportunities. At the same time, the more the local residents are involved in the event, the more they are likely to experience the excitement of the event. Residents with a high degree of involvement have a strong sense of cultural identity and expression of the value of the event and are more likely to gain confidence, respect, and friendship through enhanced communication. All of these associations with the event (engagement) reinforce the positive influence of positive emotions on residents’ attitudes toward the event.

In addition, the moderating effect of involvement has a more obvious impact on low-level residents. The main reason may be that the residents in the high-involvement group have more diverse channels and types of information to follow and obtain, and have a more comprehensive and deeper evaluation of the event. While positive information may deepen the relationship between positive feelings and attitudes, the negative information will weaken that relationship. The low-involvement residents are more susceptible to emotional factors due to limited information. This also validates Zaichkowsky’s (1985) view that products with high involvement are evaluated by quality, while products with low involvement are more susceptible to emotion.

Overall, the results of this study confirmed that involvement would positive moderate the relationship between psychic income and supportive attitude, and further proved that the involvement of the residents in sports events is an important influencing factor of psychic income and attitude as suggested by other scholars in their studies (Kim and Walker, 2012; Gibson et al., 2014; Liu, 2017). Therefore, it is suggested that the host city government and event organizers should involve local residents in the decision-making process about sports events bidding, planning, and organizing, address the concerns of the residents, and provide more opportunities for residents’ interaction and participation, so as to win their support toward the event.

Based on our study, we derive specific implications:

(1) Promoting media publicity, especially online information communication, and other online channels, adhering to the systematic, normalized, and civic publicity. Full coverage of publicity and report can be reached through systematic reports from the knowledge popularization of the sports events and projects, to the experience of events undertaking and services; from the mobilization of public participation and volunteer services to the construction of venues and reserve forces; from the actual events to the development and change of the city and relating industries. Combined with specific offline work, we must ensure that in the hosting of the brand events, residents can have the right to know, the right to participate, the right to make decisions, and the right to gain from the events, and in turn, guide residents to have the “sense of ownership” in the whole process of the construction of brand events.
(2) Actively exploring and developing theme activities, publicity activities, educational activities, training activities that can continue the different periods of the events and in association with the city’s positioning, culture, and goal; strengthening the guidance of residents’ active participation of multiple stages as in the pre-event planning stage, on-going development of the event and post-event utilization stage. An association between the overall health of public and sports culture can be made through the combination between the sports events and citizens’ healthy lifestyles, which makes the profound culture and value of the events highly agree with the proposal of national fitness and healthy living, integrating the sports events with the many essential ways of residents’ daily living, enhancing deeper involvement and in turn bringing the residents joy and happiness in an even deeper level.
(3) More work is required in the research of sports, especially the investigation and research relating to the events. In addition, specific sports services by grass-root units like online public opinion analysis, public hotline, project association, events venues, and street communities are also helpful in understanding the central appeal and need of local residents of the host city in various channels and angles. Moreover, it is important that the development of the sports events and residents’ needs are connected so that local residents can have more opportunities to participate, interact and express their opinions in the events.

## Limitations and Future Studies

Firstly, the selection of the case has its limitation: this model only tested and verified the Shanghai Masters. Despite research indicating that this model can be applied to other similar sports events or used as a reference, the explanatory power of the research conclusions may be insufficient when the types of cases change. In future research, more cases can be included, and relevant research on other different categories of events can be carried out to gain more empirical experience, and even empirical research on different cities and different events scales can be considered to enrich the model and scale content.

Secondly, acquisition of the data for measurement has its time limit: this research chose the approach of pre-event research and failed to conduct a tracing study after the event due to the limited resources and the actual situation of the event being held over the past years, and as a result, the capability of verifying whether or not the psychic income model of such events will vary according to the different period of the event is limited. In the subsequent research, we can consider including tracing research, obtaining research data from the pre-event, in-event, and post-event period, and dynamically and comprehensively analyzing the change of residents’ psychic income in the sports events, so that more sophisticated proofs can be contributed to the improvement of the policies and services of the sports event in different stages.

In addition, this study only used involvement as the moderating variable and other influencing factors, such as gender, age, length of residence could be included in future studies.

## Data Availability Statement

The raw data supporting the conclusions of this article will be made available by the authors, without undue reservation.

## Author Contributions

FZ had the idea and completed the research content and full-text writing. DL participated in the work of research ideas and research methods. DP completed the translation of the full text. MC completed the proofreading, typesetting, and format adjustment. All authors contributed to the article and approved the submitted version.

## Conflict of Interest

The authors declare that the research was conducted in the absence of any commercial or financial relationships that could be construed as a potential conflict of interest.

## Publisher’s Note

All claims expressed in this article are solely those of the authors and do not necessarily represent those of their affiliated organizations, or those of the publisher, the editors and the reviewers. Any product that may be evaluated in this article, or claim that may be made by its manufacturer, is not guaranteed or endorsed by the publisher.
